# Level of dengue preventive practices and associated factors in a Malaysian residential area during the COVID-19 pandemic: A cross-sectional study

**DOI:** 10.1371/journal.pone.0267899

**Published:** 2022-04-29

**Authors:** Dina Nurfarahin Mashudi, Norliza Ahmad, Salmiah Mohd Said

**Affiliations:** 1 Department of Community Health, Faculty of Medicine and Health Sciences, Universiti Putra Malaysia, Selangor, Malaysia; 2 Ministry of Health Malaysia, Putrajaya, Malaysia; National Taiwan University, TAIWAN

## Abstract

**Background:**

Dengue fever is a mosquito-borne viral infection that is endemic in more than 100 countries and has the highest incidence among infectious diseases in Malaysia. The increase of dengue fever cases during the COVID-19 pandemic and the movement control order (MCO) highlighted the necessity to assess the dengue preventive practices among the population. Thus, this study aimed to determine the level of dengue preventive practices and its associated factors among residents in a residential area in Johor, Malaysia during the COVID-19 pandemic.

**Method:**

A community-based cross-sectional study was conducted on 303 respondents from a Johor residential area between May and June 2021. A validated self-administered questionnaire was created using google forms and distributed to the respondents via WhatsApp. The questionnaire consisted of three sections: (i) Sociodemographic characteristics and history of dengue fever, (ii) dengue preventive practices, and (iii) six constructs of the Health Belief Model (HBM). The association between the dependent and independent variables were examined using multiple logistic regression with a significant level set at less than 0.05.

**Result:**

About half of the respondents have a good level of dengue preventive practices. Respondents with a history of dengue fever (aOR = 2.1, 95% CI: 1.1–4.2, p = 0.033), low perceived susceptibility (aOR = 1.8, 95% CI: 1.1–3.0, p = 0.018), high self-efficacy (aOR = 1.7, 95% CI: 1.0–2.8, p = 0.045), and high cues to take action (aOR = 2.5, 95% CI: 1.5–4.2, p < 0.001) had higher odds of practicing good dengue preventive measures.

**Conclusion:**

This study demonstrated a moderate level of dengue preventive practices during the COVID-19 pandemic. Therefore, a stronger dengue control programme is recommended by focusing on cues to take action, self-efficacy, and recruiting those with a history of dengue fever to assist health authorities in promoting good dengue preventive practices in the community.

## Background

Dengue is regarded as the most eminent mosquito-borne viral disease by the World Health Organization (WHO) [1]. Dengue can infect a person up to four times as there are reportedly four types of dengue viruses that can cause dengue infection [2]. The disease is currently endemic in more than 100 countries throughout the WHO regions, with Asia accounting for more than half of the worldwide burden [2]. In Malaysia, dengue fever has the highest incidence rate among communicable diseases, with 397.71 cases per 100,000 populations [3]. Dengue fever is generally an urban disease, and the three Malaysian states with the highest number of recorded cases are Selangor, Wilayah Persekutuan Kuala Lumpur, and Johor [4].

Besides dengue fever, the Coronavirus Disease 2019 (COVID-19), which was caused by Severe Acute Respiratory Syndrome Coronavirus 2 (SARS-CoV-2) [5], had a global and Malaysian impact. The COVID-19 was declared a Public Health Emergency of International Concern (PHEIC) by WHO on January 30, 2020 [6]. In addition to the predicaments posed by COVID-19, dengue-endemic countries must deal with the rise of dengue cases [7, 8]. The movement control order (MCO) enforced during the COVID-19 pandemic in Malaysia had an impact on the incidence of dengue fever [9, 10]. A simulation study based on a previously reported agent-based model found that dengue cases were significantly higher during the lockdown and worsened during partial lockdown [11]. Dengue fever cases in Johor Bahru grew from 4400 in 2018 to 8105 in 2019, and then 9412 cases in 2020. In 2021, there were 998 dengue cases reported in Johor Bahru as of June [12]. According to the epidemiological week from 2018 to 2020, dengue cases inclined from week 13 (25th March 2020) consistent with the MCO implementation commenced on 13th March 2020. In Taman Kota Masai, Johor Bahru, Johor, the number of dengue cases reported before the pandemic was 36 cases in 2018, 228 in 2019, and during the pandemic COVID-19, the dengue cases reported increased to 417 dengue cases in 2020 [12].

Dengue fever is a serious threat to the population. Aside from the clinical complications of the disease, [13] dengue fever have a significant impact on the family’s economics and subsequently the general society [14–16]. The situation is aggravated by the co-circulation of dengue viruses and SARs-Cov-2, which creates a greater effect on an individual and causes delays in diagnosis and treatment. It also affects public health control, overwhelms the healthcare system, causes underreporting and disintegration in surveillance and control intervention [7]. The dramatic change in human mobility following the COVID-19 pandemic is also likely to shift the dengue transmission [17].

Previous studies have shown that using mosquito repellent, covering water containers with lids, removing stagnant water, and using insecticide are all effective dengue preventive practices [18–22]. According to studies on the level of dengue preventive practices, less than 80% of respondents practised moderate to good dengue preventive measures and Health Belief Model (HBM) can be used to predict it [23–26]. The HBM is among the oldest theories widely used as a social cognition model in predicting health behaviour [26]. According to HBM, an individual’s commitment to health-promoting behaviour is influenced by their views about the severity of the health problem and their likelihood of contracting the disease, as well as their perceptions of the benefits and barriers to the health behaviour [27]. Studies have used HBM to predict dengue preventive practices [25, 26].

Furthermore, both Aedes aegypti and Aedes albopictus are highly anthropophilic, preventive actions are especially important during the COVID-19 pandemic as indoor mosquitoes increased during the lockdown [28]. In addition, having a history of dengue fever may play a vital role in dengue preventive actions, but earlier researchers have found inconclusive associations [25, 26]. Therefore, this study aimed to identify the level of dengue preventive practices and its associated factors during the COVID-19 pandemic using the theoretical construct of HBM. We hypothesised that good dengue preventive practices are associated with a higher perception of getting dengue, their beliefs on the severity of dengue, and their corresponding with their perceptions towards the benefits of and barriers to the dengue preventive practices. It is also associated with higher cues to take action and confidence in performing dengue preventive practices.

## Methods

### Study design and setting

A cross-sectional study was conducted in Taman Kota Masai, a residential area in Johor Bahru district under Pasir Gudang Municipal Council. Johor Bahru is the capital of the state of Johor, located at the southern end of Peninsular Malaysia. The Malay ethnicity group makes up the majority (74.72%) of the population in Pasir Gudang, followed by Indians (4.62%) and Chinese (2.33%) [29]. Johor is the state with the highest reported dengue cases after Selangor and Kuala Lumpur. Nevertheless, Johor has a limited number of studies compared to the other two states. Johor Bahru district recorded the most cases of dengue fever in Johor, accounting for almost 80% of all the cases in the state. Taman Kota Masai was chosen as the study location because it is one of the residential areas with frequent dengue outbreaks, with approximately 100 incidents in 2020 [12]. The Ministry of Health divided Taman Kota Masai into 35 zones to maximise vector control in the localities. The population of Taman Kota Masai is estimated at 92 thousand people. Terrace houses make up the majority of the housing areas, which are surrounded by other residential and industrial regions. Data were collected from May to June 2021. This study adhered to the Strengthening the Reporting of Observational Studies in Epidemiology (STROBE) guidelines for cross-sectional epidemiological studies in terms of design, setting, analysis, and reporting (see S1 Table for the detailed checklist of STROBE criteria).

### Sampling and sample size

The required number of samples for this study was calculated using two independent gender proportions based on Rakhmani (2018) [24] and the formula by Lemeshow et al. (1990) [30]. The final sample size needed for this study was 646 after adjusting for the estimated sample design effect for cluster sampling where the effect size was two and assuming a 60% response rate.

The sampling method used was two-stage cluster sampling. In the first stage, the zones were selected from 35 existing areas. However, five zones were omitted as they were located in the factory or the new residential area. Thus, 16 over 30 zones were randomly selected using a random number generator. Next, roads were chosen from each of the zones. The number of roads chosen for each zone was determined by the zone’s total number of roads, where more samples were collected in the zone with a greater number of roads. From the 16 zones, only one zone contributed three roads, while the rest contributed one road each. The roads were also randomly selected using a random number generator. Thus, a total of 18 roads were selected from the study area. From the chosen roads, the heads of households who were over 18 years old, had resided in Taman Kota Masai for more than 6 months, and were able to communicate through WhatsApp application were recruited for this study. The household heads were chosen because they symbolise the behaviour of the household and influence health practices [31]. Moreover, vector control at the household level is a fundamental strategy for dengue fever prevention [32, 33]. The WhatsApp application was used as the communication medium to minimise contact during the MCO, and it is one of the widely used forms of communication among Malaysian adults [34].

### Ethics approval and consent to participate

Ethical approval was obtained from Ethics Committee for Research Involving Human Subject University Putra Malaysia, and the referral number is UPM/TNPCI/RMC/1.4.18.2(JKEUPM). Implied consent was obtained from respondents via a google form before participation in this study. The identity of the respondents are kept anonymous, and the information in this study is kept strictly confidential.

### Study instrument

The questionnaire used in this study was adapted from previous studies [35–38]. The content validity of the questionnaire was reviewed by an expert panel composed of public health specialists. The questionnaire was modified based on the experts’ comments before being checked for face validity. The questionnaire’s face validity was determined by asking five respondents who did not participate in the main study to provide comments on the questionnaire’s sentence, wording, and structure. Their comments on the comprehensibility, language suitability, and duration to complete the questionnaire were also taken into consideration. Later, the questionnaire was amended based on their comments. A test-retest was conducted among 30 residents in other localities in Johor Bahru to examine the stability of the questionnaire. The data from this assessment was not included in the final analysis. The data was analysed using intra-class correlation coefficient (ICC), and the ICC ranged between 0.69–0.962, which were within good to excellent agreement [39, 40]. Data was collected online using Google Forms, which was distributed to the respondents via the WhatsApp application. The self-administered online questionnaire was in the Malay language, the national language of Malaysia and consisted of three parts:

Part A–Sociodemographic data and history of dengue fever

Part B–Dengue preventive practices

Part C–Constructs of Health Belief Model (HBM)

#### Dependent variable

The dependent variable in this study was the level of dengue preventive practices, which was adapted from a validated questionnaire with a Cronbach’s alpha value of 0.790 [36]. Dengue preventive practices are defined as respondents’ efforts to avoid contracting dengue fever and preventing action to minimize dengue fever occurrence [36]. The preventive practices measured in this study include prevention of mosquito breeding, prevention of mosquito bites and prevention of dengue transmission [26]. Respondents were asked to rate 15 items on a five-point Likert scale, i.e., “never”, “rarely”, “sometimes”, “usually”, and “always”, with scores “1” to “5”, respectively. The total score ranged from 15 to 75. The scores were dichotomised for analysis using the median split, with 15–59 being categorised as poor practice and 60–75 as good practice.

#### Independent variable

The sociodemographic profile of respondents that included age, gender, highest education level, and monthly household income was collected. For analysis, age was divided into ≤ 30 years old (youth) and > 30 years old [41]; the educational level was categorised into primary, secondary, and tertiary; the monthly household income was classified into ≤ MYR 4,849, which represents the bottom 40% of population income (B40), and income of > MYR 4,849 [42]. The previous history of dengue fever was assessed by asking the respondents or their family members if they had been admitted to the hospital due to dengue, with answers ranging from “yes”, “no”, or “unsure”.

The constructs of HBM include perceived susceptibility, perceived benefit, perceived barrier, perceive severity, self-efficacy and cues to take action. The definition of these constructs are as follows; perceive susceptibility is defined as assessing one’s subjective perception of the risk of getting dengue; perceive benefit is defined as if they believe that a dengue prevention practice would reduce the susceptibility or severity or lead to another positive outcome; perceive barrier define as if they perceive few negative attributes related to dengue prevention practice; perceive severity is one’s belief of how severe the condition is and its consequences; self-efficacy is defined as the opinion of an individual to perform dengue preventive practices successfully, and cues to take action against dengue vectors are defined as things that may affect the individual’s perception and may indirectly influence their health-related behaviour [23, 26, 43]. The items on the HBM constructs, which comprised of perceived susceptibility, benefits, and severity of dengue were adopted from a study with the Cronbach’s alpha values of 0.943, 0.910, and 0.591, respectively [37]. The perceived barrier, cues to take action against dengue vector, and self-efficacy were taken from two earlier studies in Selangor, Malaysia [35, 38]. No Cronbach’s alpha value were reported.

The assessment of perceived benefit consisted of five items rated using a four-point Likert scale ranging from “strongly disagree” (1) to “strongly agree” (4) with a total score range of 5–20. The perceived benefit was categorised into low (5–17) and high (18–20) using a cut-off point determined by the median split. Meanwhile, perceived susceptibility consisted of six items that examined the respondents’ perception of the risk of getting dengue fever using a similar four-point Likert scale. Four items (C3.1; C3.2, C3.3, and C3.4) had a reverse four-point Likert scale coding ranging from “strongly disagree” (4) to “strongly agree” (1). The total scores were between 6 and 24, with 18–24 was classified as high perceived susceptibility and 6–17 as low.

Perceived severity consisted of four items examining the respondents’ feelings towards the seriousness of dengue fever. The score used a four-point Likert scale and scores added up to 4–16. Only item C4.3 had a reverse coding in this construct. The scores of 15–16 were categorised as having high perceived severity and 4–14 as low perceived severity. The perceived barrier also used a four-point Likert scale to answer six questions on barriers in performing dengue prevention practice. The range of scores was 6–24. Those scoring 6 to 11 have a lower perceived barrier, whereas those scoring 12 to 24 have a higher perceived barrier.

Nine items made up the cues to take action against the dengue vector construct and three items made up self-efficacy. The total score from a four-point Likert scale for cues to take action was 9–36. The scores 30–36 were categorised as having high cues to take action, and scores 9–29 were categorised as low cues to take action. For self-efficacy, respondents who answered “strongly agree” and “agree” to questions C6.1 and C6.3 and “strongly disagree” and “disagree” to question C6.2 were considered confident in performing dengue preventive practices.

### Statistical analysis

The data collected was analysed using Statistical Package for Social Sciences (SPSS) version 25.0 (IBM Corp, 2016). Data were cleaned prior to the data analysis and data for each continuous variable was checked for normal distribution. Descriptive statistics were performed on the sociodemographic factors, history of dengue fever, levels of dengue prevention practice, perceived susceptibility to dengue fever, perceived benefit of preventive practices, perceived barriers, perceived severity of dengue fever, cues to take action against dengue vector, and self-efficacy variables and were presented in frequency and percentage for categorical data. The median and interquartile range was used for data that were not normally distributed.

Simple and multiple logistic regression were used to determine the associations between the sociodemographic factors, history of dengue fever, perceived susceptibility, perceived benefit, perceived barrier, perceived severity of dengue fever, cues to take action against dengue vector, and self-efficacy with the level of dengue preventive practices. The results were expressed as crude and adjusted odds ratios with the statistical significance level set at less than 0.05 (p < 0.05).

## Result

### Participation rate

A total of 646 eligible respondents from Taman Kota Masai were invited to participate in this study, but only 303 sets of questionnaires were completed, giving a response rate of 47%. Despite the low response rate, the post-hoc power analysis of this study yielded 80.3% power, indicating that the result of this study has sufficient power to detect statistical differences [44]. The parameters used to calculate post-hoc power analysis of comparing two independent groups for this study were between respondents with previous history of dengue fever and no previous history of dengue fever.

### Characteristics of the respondents

Table 1 illustrates the sociodemographic characteristics of the household head and the history of dengue fever illness of the household head and their family members. Almost all the respondents were Malays (99.4%). There were more female (53.1%) than male (46.9%) respondents with a median age of 49 years old and an interquartile range of 14. The majority of the head of the households had secondary as the highest education level (55.4%), and a monthly household income of ≤ MYR 4,849.00 (B40) [42]. About one-fifth of either the head of household or their family members had a history of dengue fever.

**Table 1:** Sociodemographic characteristics of the head of household and history of dengue fever.

Sociodemographic factors	Frequency (%)	Median (IQR)
**Age (years)**		49 (14)
• 18–30 years old	20 (6.6%)	
• 31–40 years old	55 (18.2%)	
• 41–50 years old	112 (37.0%)	
• 51–60 years old	105 (34.7%)	
• >60 years old	11 (3.6%)	
**Gender**		
• Female	161 (53.1%)	
• Male	142 (46.9%)	
**Educational level**		
• Primary	18 (5.9%)	
• Secondary	168 (55.4%)	
• Tertiary	117 (38.6%)	
**Monthly household income (Ringgit Malaysia/MYR)**		3000 (3000)
• ≤ MYR 4,849	214 (70.6%)	
• > MYR 4,849	89 (29.4%)	
**Previous history of dengue fever among respondents**		
• Yes	50 (16.5%)	
• No	249 (82.2%)	
• Not sure	4 (1.3%)	
**Previous history of dengue fever among family members**		
• Yes	67 (22.1%)	
• No	234 (77.2%)	
• Not sure	2 (0.7%)	

IQR = interquartile range.

### Level of dengue preventive practices

Only approximately half of the respondents performed good dengue preventive practices (50.2%). The maximum score for dengue preventive practices was 75, and the minimum score was 15 with a median of 60 and an interquartile range of 9. The most common dengue prevention practices performed by the participants were the use of a water container with a lid. 84.49% of the respondents usually and always use water containers with lids and close them immediately after usage, and 81.85% of them usually and always clean the water container when they found mosquito larvae. 55.78% of respondents usually and frequently change the water in the vase cover, and 56.11% of them check the presence of mosquito larvae in the vase cover. Mosquito repellent and mosquito nets usage are the least common dengue prevention measures reported by participants. Only 43.23% of the respondents usually and always use mosquito repellent, and 4.95% stated to use a mosquito net.

### Level of HBM constructs among respondents

More than half of the respondents had high levels of perceived susceptibility, perceived benefit, perceived barrier, perceived severity, cues to take action, and self-efficacy to execute dengue preventive practices. The scores and the respective constructs of the HBM are shown in Table 2.

**Table 2:** Scores and level of perceived susceptibility, perceived benefit, perceived barrier, perceived severity, self-efficacy, and cues to take action.

Variables	Minimum Score	Maximum score	Median (IQR)	Frequency (%)
**Perceive susceptibility**	9	24	18 (4)	
• Low				143 (47.2%)
• High				160 (52.8%)
**Perceive benefit**	5	20	18 (4)	
• Low				136 (44.9%)
• High				167 (55.1%)
**Perceive barrier**	6	21	12 (5)	
• Low				133 (43.9%)
• High				170 (56.1%)
**Perceive severity**	7	16	15 (2)	
• Low				148 (48.8%)
• High				155 (51.2%)
**Cues to take action**	17	36	30 (5)	
• Low				149 (49.2%)
• High				154 (50.8%)
**Self-efficacy**	7	12	9 (1)	
• No				140 (46.2%)
• Yes				163 (53.8%)

IQR = interquartile range.

### Associations of good dengue preventive practices

Through simple logistics regression analysis, this study found that people with a history of dengue fever, high perceived benefit, perceived barriers, cues to take action, and self-efficacy were significantly associated with a good level of dengue preventive (Table 3). Meanwhile, sociodemographic factors were not significantly associated with the dengue preventive practices. However, multiple logistic regression analyses revealed that only history of dengue fever, perceived susceptibility, cues to take action, and self-efficacy were significantly linked to a good level of dengue prevention. Respondents with prior experience of dengue fever were twice more likely to have good dengue preventive practices compared to individuals who had no history of dengue fever (aOR = 2.4, 95% CI: 1.2–4.7, p = 0.012). Additionally, respondents with low perceived susceptibility were nearly twice more likely to have strong dengue preventive measures than those with high perceived susceptibility (aOR = 1.8, 95% CI: 1.1–3.0, p = 0.018). Respondents with high cues to take action were 2.6 times more likely than those with low cues to take action to have a good level of dengue preventive practices (aOR = 2.6, 95% CI: 1.6–4.2, p < 0.001). Also, respondents with yes self-efficacy were nearly twice as likely as those with no self-efficacy to have good dengue preventive practices (aOR = 1.8, 95% CI: 1.1–2.9, p = 0.023).

**Table 3:** Associations of good dengue preventive practices among residents in Taman Kota Masai.

Variables	Simple Logistic Regression		Multiple Logistic Regression					
	Crude OR	P value	Coefficient	Adjusted OR	SE	P value	95% CI	
							Lower	Upper
**Intercept**			-1.201	0.3	0.267	< 0.001		
**History of dengue fever**								
• No	Ref.		Ref.					
• Yes	2.4	0.007*	0.875	2.4	0.345	0.012*	1.213	4.697
**Family history of dengue fever**								
•	Ref		Ref					
• Yes	1.6	0.078						
**Perceived susceptibility**								
• High	Ref		Ref					
• Low	1.5	0.095	0.595	1.8	0.252	0.018*	1.106	2.971
**Perceived benefit**								
• Low	Ref		Ref					
• High	1.8	0.010*						
**Perceived barriers**								
• Low	Ref		Ref					
• High	0.6	0.018*						
**Perceived severity**								
• Low	Ref		Ref					
• High	1.5	0.096						
**Cues to take action**								
• Low	Ref.		Ref					
•	2.9	< 0.001*	0.963	2.6	0.246	< 0.001*	1.616	4.243
**Self-efficacy**								
• No	Ref.		Ref					
• Yes	1.8	0.010*	0.573	1.8	0.253	0.023*	1.081	2.912

*Significant level at p < 0.05. Only significant variables were shown for multivariate logistic regression.

## Discussion

This study was conducted to evaluate the level of dengue preventive practices and its associated factors during the COVID-19 pandemic among residents of Taman Kota Masai, Johor. The result showed that only half of the respondents demonstrated a good level of dengue preventive practices. It is slightly lower than the results of a study conducted in another Malaysian state with dengue hotspot areas before the pandemic COVID-19. The study reported that 56% of the population practised good dengue prevention [45]. It shows that even people stay at home during the lockdown, the level of dengue prevention is still low. Moreover, a study that compared dengue preventive practices among communities lived in hotspot and non-hotspot dengue areas in Selangor discovered that more people in non-hotspot areas engaged in dengue preventive practices [46]. Hence, the low level of dengue prevention practices in Taman Kota Masai may be related to people’s health beliefs rather than the amount of time they spent at home during a pandemic.

In terms of health belief using the HBM, this study showed that respondents with a low perceived susceptibility were nearly twice more likely to have a good level of dengue preventive practices than those with high perceived susceptibility. A plausible explanation for this may be that people who practise good dengue prevention measures such as cleaning their house area have a reduced perception of susceptibility to contract dengue fever [47]. A study showed that people had higher health awareness during the pandemic COVID -19 [48]. They practice hygiene to a high degree, including dengue prevention. In addition, most people avoid visiting hospitals or health facilities during the pandemic, and the study also showed that the use of personal health care abruptly decreased [49]. Fear of contracting COVID -19 causes patients to avoid visiting health care facilities. It prompts them to do their utmost to stay healthy, including implementing dengue prevention measures to reduce their susceptibility to contracting dengue fever.

Furthermore, this study showed that respondents with high cues to take action were more than twice likely to have a good level of dengue preventive practices compared to those with low cues to act. Cues to take action might be internal or external, ranging from personal experiences, cultural schemas, and information received from other sources or networks [50, 51]. There is currently a lack of study examining the association between cues to take action and dengue preventive practices. According to some researchers, this construct may fade or be volatile [43, 52]. Most of the study participants agreed that they would take precautionary measures if their residential area was declared a dengue hotspot area and were constantly reminded to carry out dengue fever preventive measures by the local authorities. In the COVID-19 pandemic, the residents spent more time at home during MCO. Thus, this will allow them to be more aware of all the banners put by the health authorities concerning dengue preventive practices as their locality is a dengue hotspot area. This is the cue for them to perform the dengue preventive practices. Besides, during MCO, certain people will follow the trend and tend to change their interest such as being involved in gardening [53, 54], which also can be the cue for them to be indirectly involved in cleaning their gardening area and mosquito breeding area such as cleaning the vase cover. Additionally, a randomised control trial conducted in a different Malaysian state suggested that alerting residents when dengue positive mosquitoes were found during surveillance activities could improve source reduction practices [55]. A qualitative study conducted in two villages in Central Java province, Indonesia showed that continual media campaigns were relevant to the improvement of dengue preventive practices [56]. Hence, this demonstrates the importance of publicising hotspot areas and implementing new proactive strategies, such as notifying the community about positive dengue mosquitoes findings to maintain cues to act. Health education initiatives should also be performed regularly, especially during pandemics, to ensure sustainable cues to take action for dengue preventive practices.

In addition, respondents with self-efficacy (confidence to perform dengue preventive practices) were almost twice as likely to have a good level of dengue preventive practices compared to those with no self-efficacy. This finding is corroborated by a previous study that demonstrated self-efficacy to be a predictor of dengue preventive measures [25]. Self-efficacy is an important construct in the HBM that encourages individuals to implement preventive practices [57]. The lack of self-efficacy is one of the challenges that must be overcome to effectively apply these initiatives [23]. Accordingly, authorities should regularly deliver clear messages and demonstrate simple dengue preventive practices to boost people’s confidence and enhance self-efficacy [38, 58]. Namely, the health authority may produce a short educational video with trained role-players demonstrating the recommended practices. The video could be distributed to those with low confidence in undertaking dengue preventive activities, with the request to practise the approaches until full confidence is achieved [58]. During the COVID-19 pandemic, a study shows a surge in social media use [59]. Personal and professional lives merged through platforms like Facebook, Twitter, and Instagram, united in isolation throughout MCO Twitter is used to create global knowledge networks by facilitating academic discussions and information sharing through crowdsourcing [60]. The hashtag is also becoming increasingly popular in the online medical community to interact and share best practices. Consequently, the snappy tweets and infographics with relevant data about dengue can strengthen readers’ self-efficacy in implementing significant dengue preventive practices.

Aside from that, having a history of dengue fever was found to be significantly associated with dengue preventive practices in this study. This is in line with a study conducted among international students at a public university in Malaysia, which found that students who had previously contracted dengue fever had a good level of dengue preventive practices. The authors suggested that experience contributed to patients’ increased knowledge and awareness [61]. Those with a history of dengue fever may have obtained information on dengue fever prevention during a consultation with healthcare providers. Patients may also actively seek information after contracting dengue fever. This is in accord with a study conducted among Malaysians aged more than 18 years old, where respondents with a history of dengue fever had significantly higher knowledge about dengue fever and dengue preventive practices [26]. Thus, it was proposed that people who have experienced dengue fever be recruited to assist health authorities to promote dengue preventive practices in the communities.

## Strength and limitations of the study

This study captured dengue preventive practices at the household level and may contribute to added knowledge on dengue preventive practices during the pandemic of other infectious diseases such as COVID-19 and can be used as baseline data in planning specific health intervention strategies in the future.

The response rate of this current study was 47%, which is considered satisfactory given that some studies reported a 43% response rate for online surveys [62]. During the data collection process, a variety of methods to boost response were used, such as using a push survey in which the respondents were sent a direct link to the Google Forms questionnaire, three reminders, enlisting the help of communities leaders to remind the communities, and convincing the respondents that their opinion is valuable to eradicate dengue. Also, the researcher extended the data collection deadline to 14 days. Regardless, a post-hoc power analysis revealed that the power of this study is adequate to detect the statistically significant differences.

Conventional methods involving face-to-face interaction are not feasible during the COVID-19 pandemic. Consequently, the data collection was conducted solely through Google Forms that were disseminated via WhatsApp, which may have caused hesitancy and suspicion among the respondents. Respondents might be unable to differentiate between spam messages and legitimate research work [63]. Moreover, selection bias might occur whereby elderly persons who might not be familiar with google form may be left out. This might explain median age of the respondents was at 49 years. The use of only national language in the google form may have resulted in the majority of the respondents being Malays. Furthermore, the use of a self-reported questionnaire in this study may cause social desirability bias.

## Conclusion

A good level of dengue preventive was practised by around half of the respondents in this study. The previous history of dengue fever, low perceived susceptibility, high self-efficacy and cues to take action were the factors found to be associated with a good level of dengue preventive practices during the COVID-19 pandemic.

This study recommended that people with dengue fever experience be recruited to help promote dengue preventive practices in the communities. Additionally, cues to act can be encouraged by disclosing hotspot areas and developing new proactive strategies, such as informing communities on positive dengue mosquitoes’ findings. Besides, health education initiatives to prevent dengue must be done routinely, including during the pandemic, to ensure long-term actions and enhanced self-efficacy. The health authorities should also provide direct information and demonstrate simple dengue preventive practices regularly.

## Supporting information

S1 Table Completed of STROBE checklist of the study. (PDF)

S2 Table Frequency and percentage of Health Belief Model. (PDF)

S1 File Data of the study. (XLSX)
