# Supplementary material for: Level of dengue preventive practices and associated factors in a Malaysian residential area during the COVID-19 pandemic: A cross-sectional study
Source: PLoS One. 2022 Apr 29;17(4):e0267899. doi: 10.1371/journal.pone.0267899 (PMC9053802; doi:10.1371/journal.pone.0267899)
Supplement: S1 Table — (PDF) [file pone.0267899.s001.pdf]

STROBE Statement - Checklist of items that should be included in reports of cross-sectional studies

Items	Item No	Recommendation	Subheading of article
**Title and abstract**	1	(a) Indicate the study's design with a commonly used term in the title or the abstract<br>*Level of dengue preventive practices and associated factors in a Malaysian residential area during the Covid-19 pandemic: A cross-sectional study*	*Title*
		(b) Provide in the abstract an informative and balanced summary of what was done and what was found<br>*Abstract in this study consisting of background, method, result and conclusion sections with informative and balanced information.*	*Abstract*
**Introduction**			
Background/rationale	2	Explain the scientific background and rationale for the investigation being reported<br>*We provided specific background related to the important of dengue fever (DF) in public health, increasing number of dengue fever during COVID-19 pandemic. We stated in the end of Background section: "[18-22]. According to studies on the level of dengue preventive practices, less than 80% of respondents practised moderate to good dengue preventive measures and Health Belief Model (HBM) can be used to predict it. Since both Aedes aegypti and Aedes albopictus are highly anthropophilic, preventive actions are especially important during the COVID-19 pandemic as indoor mosquitoes increased during the lockdown. Furthermore, having a history of dengue fever may play a vital role in dengue preventive actions, but earlier researchers have found inconclusive associations." Due to this gap, thus the study was conducted.*	*Background*
Objectives	3	State specific objectives, including any prespecified hypotheses (N/A)<br>*"Therefore, this study aimed to identify the level of dengue preventive practices with the coexistence of COVID-19 and its associated factors using the theoretical construct of HBM"*	*Background*
**Methods**			
Study design	4	Present key elements of study design early in the paper<br>*This study was cross-sectional study. "A cross-sectional study was conducted in one of the residential areas in Johor Bahru district which is Taman Kota Masai"*	*Study design and setting*
Setting	5	Describe the setting, locations, and relevant dates, including periods of recruitment (N/A), exposure (N/A), follow-up (N/A), and data collection<br>*Setting of study: The head of household reside in Taman Kota Masai, Johor Bahru that meet inclusion criteria were interview after informed concerned. Locationsof study: "Johor Bahru district recorded the most cases of dengue fever in Johor, accounting for almost 80% of all the cases in the state. Taman Kota Masai was chosen as the study location because it is one of the residential areas with frequent dengue outbreaks, with approximately 100 incidents in 2020". Relevant dates of study or data collection: "Data were collected from May to June 2021."*	*Study design and setting.*
Participants	6	(a) Give the eligibility criteria, and the sources and methods of selection of participants<br>*Eligible criteria in this study: "From the chosen roads, the heads of households who were over 18 years old, had resided in Taman Kota Masai for more than 6 months, and were able to communicate through WhatsApp application were recruited for this study" The required number of samples was calculated from using two independent proportions for gender in the study by Rakhmani (2018) using the formula by Lemeshow (1990). After adjustment, the final sample size required for this study is 646 residents in Taman Kota Masai."*	*Sampling and sample size*

Items	Item No	Recommendation	Subheading of article
Variables	7	Clearly define all outcomes, exposures (N/A), predictors, potential confounders, and effect modifiers (N/A). Give diagnostic criteria (N/A)<br>*Dependent variables or outcome of the study: Level of dengue preventive practices. Independent variables or predictors in this study: age, gender, educational status, monthly household income in Ringgit Malaysia (MYR), whether or not they or family members had already suffered from DF, and six constructs of Health Belief Model. "Simple and multiple logistic regression were used to determine the associations between the sociodemographic factors, history of dengue fever, perceived susceptibility, perceived benefit, perceived barrier, perceived severity of dengue fever, cues to take action against dengue vector, and self-efficacy with the level of dengue preventive practices. The results were expressed as crude and adjusted odds ratios with the statistical significance level set at less than 0.05 (p < 0.05).*	*Dependent variables, Independent variables & Statistical analysis*
Data sources/ measurement	8*	For each variable of interest, give sources of data and details of methods of assessment (measurement). Describe comparability of assessment methods if there is more than one group.<br>*Interest variables (included dependent and independent variables) were assessed by questionnaire through Google form. "Data was collected online using Google Forms, which was distributed to the participants via the WhatsApp application. The self-administered online questionnaire was in the Malay language, the national language of Malaysia and consisted of three parts" in this study, all respondents were the head of household and the same method was used to assess variables of interest in all respondents.*	*Study instrument*
Bias	9	Describe any efforts to address potential sources of bias	
Study size	10	Explain how the study size was arrived at<br>*In this study, study size refers to sample size. The information regarding sample size is given in Participants (Item 6).*	*Sampling and sample size*
Quantitative variables	11	Explain how quantitative variables were handled in the analyses. If applicable, describe which groupings were chosen and why<br>*All variables were divided into group to give quantitative measures. One category of each variablewas used as reference category. Dependent variables (dengue preventive practices) were dichotomized into good and poor group based on median split cut-off point. These processes resulted variables become quantitative and therefore suitable for further analyses.*	*Dependent variables, Independent variables & Statistical analysis*
Statistical methods	12	(*a*) Describe all statistical methods, including those used to control for confounding<br>*"Simple and multiple logistic regression were used to determine the associations between the sociodemographic factors, history of dengue fever, perceived susceptibility, perceived benefit, perceived barrier, perceived severity of dengue fever, cues to take action against dengue vector, and self-efficacy with the level of dengue preventive practices. The results were expressed as crude and adjusted odds ratios with the statistical significance level set at less than 0.05 (p < 0.05)."*	*Statistical analysis*
		(*b*) Describe any methods used to examine subgroups and interactions	

Items	Item No	Recommendation	Subheading of article
		(*c*) Explain how missing data were addressed<br>*There was no missing data as Google form 'required' the participants to answer all of the question to complete the questionnaire*	

Items	Item No	Recommendation	Subheading of article
		(*d*) If applicable, describe analytical methods taking account of sampling strategy<br>*There is no problem related sampling strategy in our study, but the analytical analysis in this study was choose based on our data. In this study we used simple and multiple logistic regression as appropriate following the data.*	*Statistical analysis*
		(*e*) Describe any sensitivity analyses<br>*There is no any sensitivity analysis relevant to this study. However, we did questionnaire validity and reliability test "The questionnaire used in this study was adapted from previous studies and was validated by public health specialists before data collection. A test-retest was conducted among 30 residents in other localities in Johor Bahru to examine the stability of the questionnaire. The data from this assessment was not included in the final analysis."*	*Study instrument*
**Results**			
Participants	13*	(a) Report numbers of individuals at each stage of study—eg numbers potentially eligible, examined for eligibility, confirmed eligible, included in the study, completing follow-up (N/A), and analysed.<br>*In this study, "A total of 646 eligible respondents from Taman Kota Masai were invited to participate in this study, but only 303 sets of questionnaires were completed, giving a response rate of 47%.."*	*Participation rate*
		(b) Give reasons for non-participation at each stage<br>*In this study, there was no non-participant.*	
		(c) Consider use of a flow diagram	
Descriptive data	14*	(a) Give characteristics of study participants (eg demographic, clinical, social) and information on exposures and potential confounders<br>*In this study, characteristics of study participants are summarized in Table 1. We included a very little information of the Table 2 into description text to avoid repetitive.*	*Characteristics of the respondents*
		(b) Indicate number of participants with missing data for each variable of interest<br>*In this study, there was no participant with missing data.*	
Outcome data	15*	Report numbers of outcome events or summary measures<br>*The level of dengue preventive practices is described in subtopic level of dengue preventive practices.*	*Level of dengue preventive practices.*
Main results	16	(*a*) Give unadjusted estimates and, if applicable, confounder-adjusted estimates and their precision (eg, 95% confidence interval). Make clear which confounders were adjusted for and why they were included.<br>*In this study, unadjusted estimates (univariate analysis) and adjusted estimates are calculated for dependent variable and independent variable and both of them provided in Table 3.*	*Association of good dengue preventive practices*
**Items**	**Item No**	**Recommendation**	**Subheading of article**
		(*b*) Report category boundaries when continuous variables were categorized<br>*In this study, variable involving scoring were categorized into" good" or" poor", "low" or "high", and "no" or yes" based on median split cut-off point. In addition, continuous variable of age was divided into two, which were ≤30 years old (youth) and >30 years old. As for the educational level based on self-reported by respondents was interpreted into three categories: a primary, secondary, and tertiary level of education. The household income was in the form of cash for the monthly period and categorized following income classification by household by DOSM into less than RM4849 represent the B40 and rm4850 and more represent M40 and T20. These category criteria used throughout the manuscript.*	*Study variable*
		(*c*) If relevant, consider translating estimates of relative risk into absolute risk for a meaningful time period	
Other analyses	17	Report other analyses done—eg analyses of subgroups and interactions, and sensitivity analyses	

**Discussion**			
Key results	18	Summarise key results with reference to study objectives<br>*The key findings are explained throughout the discussion section with comparison with other studies.*	*Discussion*
Limitations	19	Discuss limitations of the study, taking into account sources of potential bias or imprecision. Discuss both direction and magnitude of any potential bias.<br>*Here we discussed the limitation of our study. "Conventional methods involving face-to-face interaction are not feasible during the COVID-19 pandemic. Consequently, the data collection was conducted solely through Google Forms that were disseminated via WhatsApp, which may have caused hesitancy and suspicion among the respondents. Respondents might be unable to differentiate between spam messages and legitimate research work. Furthermore, the use of a self-reported questionnaire in this study may cause social desirability bias"*	*Strength and limitation of the study*
Interpretation	20	Give a cautious overall interpretation of results considering objectives, limitations, multiplicity of analyses, results from similar studies, and other relevant evidence.<br>*Some cautious are given in the discussion related to our finding and our proposed approaches in dengue prevention program. Then we also mentioned the limitation of our study in the strength and limitation of the study.*	*Discussion*<br>*Strength and limitation of the study*
Generalisability	21	Discuss the generalisability (external validity) of the study results.	
**Other information**			
Funding	22	Give the source of funding and the role of the funders for the present study and, if applicable, for the original study on which the present article is based.<br>*Funding: Not applicable.*	*Funding*

*Give information separately for exposed and unexposed groups.

Note: An Explanation and Elaboration article discusses each checklist item and gives methodological background and published examples of transparent reporting. The STROBE checklist is best used in conjunction with this article (freely available on the Web sites of PLoS Medicine at http://www.plosmedicine.org/, Annals of Internal Medicine at http://www.annals.org/, and Epidemiology at http://www.epidem.com/). Information on the STROBE Initiative is available at www.strobe-statement.org.
