# Supplementary material for: Level of dengue preventive practices and associated factors in a Malaysian residential area during the COVID-19 pandemic: A cross-sectional study
Source: PLoS One. 2022 Apr 29;17(4):e0267899. doi: 10.1371/journal.pone.0267899 (PMC9053802; doi:10.1371/journal.pone.0267899)
Supplement: S2 Table — (PDF) [file pone.0267899.s002.pdf]

Additional file 5- Table Frequency and percentage of Likert scale answer for the construct of Health Belief Model among respondents

Construct/ item	Strongly disagree n (%)	Disagree n (%)	Agree n (%)	Strongly agree n (%)
**Perceived susceptibility**				
C3.1) The chances of me getting dengue fever are lower than other people my age	46 (15.2%)	101 (33.3%)	91 (30.0%)	65 (21.5%)
C3.2) My immune system is strong so I will not get dengue fever	81 (26.7%)	127 (41.9%)	66 (21.8%)	29 (9.6%)
C3.3) The chances of me getting dengue fever next year are low	56 (18.5%)	104 (34.3%)	92 (30.4%)	51 (16.8%)
C3.4) I believe strong and healthy people will not get dengue infection.	119 (39.3%)	123 (40.6%)	42 (13.9%)	19 (6.3%)
C3.5) I believe everyone has a chance of being infected with the dengue virus.	24 (7.9%)	17 (5.6%)	78 (25.7%)	184 (60.7%)
C3.6) I was scared if I got dengue fever.	14 (4.6%)	15 (5.0%)	57 (18.8%)	217 (71.6%)

Construct/ item	Strongly disagree n (%)	Disagree n (%)	Agree n (%)	Strongly agree n (%)
**Perceived benefit**				
C2.1) I believe cleaning residential areas from containers that hold water, such as old tires and drains around the house, can prevent mosquitoes from breeding.	3 (1%)	2 (0.7%)	26 (8.6%)	272 (89.8%)
C2.2) I believe using mosquito repellent can prevent adult mosquito bites.	7 (2.3%)	14 (4.6%)	132 (43.6%)	150 (49.5%)
C2.3) I believe using mosquito nets can prevent mosquito bites while sleeping.	9 (3.0%)	17 (5.6%)	112 (37.0%)	165 (54.5%)
C2.4) I believe fogging can kill adult mosquitoes	4 (1.3%)	15 (5.0%)	124 (40.9%)	160 (52.8%)
C2.5) I believe the use of abate in the water can kill mosquito larvae.	2 (0.7%)	8 (2.6%)	104 (34.3%)	189 (62.4%)
**Perceived barrier**				
C1.1) Fogging is dangerous to health.	86 (28.4%)	112 (37%)	79 (26.1%)	26 (8.6%)
C1.2) Abate in the water is not good for health.	56 (18.5%)	122 (40.3%)	93 (30.7%)	32 (10.6%)

Construct/ item	Strongly disagree n (%)	Disagree n (%)	Agree n (%)	Strongly agree n (%)
C1.3) I need a lot of money to implement dengue prevention at home.	135 (44.6%)	123 (40.6%)	34 (11.2)	11 (3.6%)
C1.4) My family or I feel that fogging can stain the house. So, during fog, I don't like to open doors and windows.	101 (33.3%)	106 (35.0%)	65 (21.5%)	31 (10.2%)
C1.5) The fogging in the evening was disturbing as my family and I was asked to leave the house while we were worshipping or eating.	124 (40.9%)	99 (32.7%)	50 (16.5%)	30 (9.9%)
C1.6) I have other reasons for not cooperating with dengue prevention activities.	135 (44.6%)	149 (49.2%)	17 (5.6%)	2 (0.7%)
**Perceived severity**				
C4.1) If left untreated, dengue fever will get worse	1 (0.3%)	3 (1.0%)	36 (11.9%)	263 (86.8%)
C4.2) Dengue fever can worsen to become dengue haemorrhagic fever	2 (0.7%)	5 (1.7%)	27 (8.9%)	269 (88.8%)
C4.3) Dengue fever is easily treated.	66 (21.8%)	120 (39.6%)	87 (28.7%)	30 (9.9%)
C4.4) Dengue outbreaks can occur in a place if no prevention and control measures are taken.	6 (2.0%)	5 (1.7%)	45 (14.9%)	247 (81.5%)

Construct/ item	Strongly disagree n (%)	Disagree n (%)	Agree n (%)	Strongly agree n (%)
**Cues to action**				
C5.1) I will take precautionary measures if my residential area is announced as a dengue hotspot area.	3 (1.0%)	7 (2.3%)	58 (19.1%)	235 (77.6%)
C5.2) I am constantly reminded by local authorities and health to carry out preventive measures to control dengue fever.	6 (2.0%)	5 (1.7%)	94 (31.0%)	198 (65.3%)
C5.3) The community around me does not carry out dengue prevention and control activities.	72 (23.8%)	113 (37.3%)	86 (28.4%)	32 (10.6%)
C5.4) I will help my neighbours clear the breeding grounds together.	3 (1.0%)	8 (2.6%)	109 (36.0%)	183 (60.4%)
C5.5) The announcement of information through the media on dengue fever attracted my interest to participate in dengue control/ prevention programs.	1 (0.3%)	17 (5.6%)	144 (47.5%)	141 (46.5%)
C5.6) I know how to use larva poison(abate)	11 (3.6%)	38 (12.5%)	143 (47.2%)	111 (36.6%)
C5.7) I know where to get/buy 'mosquito repellent' (mosquito repellent)	3 (1.0%)	20 (6.6%)	124 (40.9%)	156 (51.5%)

Construct/ item	Strongly disagree n (%)	Disagree n (%)	Agree n (%)	Strongly agree n (%)
C5.8) I know the potential places for mosquito breeding	2 (0.7%)	11 (3.6%)	124 (40.9%)	166 (54.8%)
C5.9) I have enough knowledge about dengue fever.	13 (4.3%)	72 (23.8%)	151 (49.8%)	67 (22.1%)
**Self-efficacy**				
C6.1) I will make sure there are no breeding grounds around my house.	1 (0.3%)	5 (1.65%)	81 (26.7%)	216 (71.3%)
C6.2) I am not sure what information needs to be passed on to my family members regarding dengue fever	70 (23.1%)	104 (34.3%)	94 (31.0%)	35 (11.6%)
C6.3) I am confident that I can practice dengue prevention and control measures well	2 (0.7%)	11 (3.6%)	116 (38.3%)	174 (57.4%)
